# A Novel ^68^Ga-Labeled Integrin α4β7-Targeted Radiopharmaceutical for PET/CT Imaging of DSS-Induced Murine Colitis

**DOI:** 10.3390/pharmaceutics17121591

**Published:** 2025-12-10

**Authors:** Guangjie Yang, Haiqiong Zhang, Li Huo

**Affiliations:** Department of Nuclear Medicine, State Key Laboratory of Complex, Severe, and Rare Diseases, Center for Rare Diseases Research, Peking Union Medical College Hospital, Chinese Academy of Medical Science and Peking Union Medical College, Beijing 100730, China; yangguangjie@pumch.cn (G.Y.); hqzhang22@163.com (H.Z.)

**Keywords:** inflammatory bowel diseases, integrin α4β7, positron emission tomography (PET), molecular imaging, dextran sulfate sodium (DSS), colitis

## Abstract

**Background**: Inflammatory bowel diseases (IBD) rely on invasive methods for detecting intestinal inflammation, with the needs for non-invasive molecular imaging tools being unmet. Integrin α4β7 is a key target in IBD pathogenesis due to its role in the recruitment of T cells. This study aimed to develop a novel ^68^Ga-labeled integrin α4β7-targeted radiopharmaceutical (^68^Ga-A2) and evaluate its feasibility for non-invasive PET/CT imaging of IBD inflammation in a dextran sulfate sodium (DSS)-induced murine colitis model. **Methods**: ^68^Ga-A2 was synthesized via radiolabeling DOTA-A2 with ^68^Ga. In vitro properties (radiochemical purity, stability, binding specificity, and affinity) of ^68^Ga-A2 were validated. The DSS-induced colitis model was established and confirmed in C57BL/6J mice, followed by in vivo PET/CT imaging, ex vivo biodistribution studies, and histological (HE and IHC) analyses to evaluate the targeting efficacy of ^68^Ga-A2. **Results**: ^68^Ga-A2 was prepared efficiently (20 min) with a radiochemical purity of >95% and demonstrated good in vitro stability. It exhibited specific binding to integrin α4β7 with a Kd of 68.48 ± 6.55 nM. While whole-body PET/CT showed no visible inflammatory focus uptake, ex vivo imaging and biodistribution of colon tissue revealed significantly higher uptake in DSS-treated mice compared to that in healthy/blocking groups, which was consistent with histological evidence of inflammation. **Conclusions**: ^68^Ga-A2 demonstrated specific targeting of IBD inflammatory foci in vitro and ex vivo. Despite whole-body imaging limitations, further optimization of its structure may enable it to become a promising non-invasive PET agent for IBD. These findings support future clinical investigations to validate its utility in IBD diagnosis and monitoring.

## 1. Introduction

Inflammatory bowel disease (IBD), including ulcerative colitis (UC) and Crohn’s disease (CD), is a chronic inflammatory condition of the gastrointestinal tract. Despite its complex and incompletely elucidated etiology, immune response dysregulation has been identified as a pivotal driver in the initiation and progression of IBD [1,2]. A key pathological feature of IBD is the aberrant recruitment and retention of activated T cells in the intestinal mucosa. Upon activation, these T cells upregulate the expression of integrin α4β7, which specifically binds to mucosal addressin cell adhesion molecule-1 (MAdCAM-1) on intestinal endothelial cells [3]. This interaction mediates T cell homing to the gut and exacerbates local inflammation. The clinical diagnosis and management of IBD currently rely on a comprehensive assessment of medical history, endoscopic findings, imaging studies, and pathological examinations [4]. However, existing diagnostic tools have notable limitations. Traditional imaging modalities such as computed tomography (CT) enterography, magnetic resonance imaging (MRI) enterography, and ultrasonography focus primarily on evaluating structural changes (e.g., intestinal wall thickening, edema) rather than molecular-level inflammatory activity, leading to low sensitivity for early-stage inflammation. Endoscopy (including colonoscopy) is considered the gold standard for early IBD diagnosis due to its ability to directly visualize mucosal lesions and obtain biopsies, but it is an invasive procedure that causes patients’ discomfort and poor compliance, particularly in long-term treatment follow-up [5]. In addition, endoscopy lacks the ability to capture the abnormalities in deep bowel wall layers and some patients may have lesions that are unreachable by colonoscope [6]. Even after diagnosis, patients still require close monitoring and endoscopic evaluation for major changes in their treatment regimen. Therefore, there is an urgent need for reliable non-invasive methods for the diagnosis, monitoring, and guidance of IBD treatment [7].

Multimodal imaging techniques such as positron emission tomography/computed tomography (PET/CT) and positron emission tomography/magnetic resonance imaging (PET/MRI) can enhance the diagnostic accuracy of IBD. Their utility goes beyond standard colonoscopy, enabling detailed visualization of areas inaccessible to endoscopy and providing a comprehensive view of inflammatory activity and structural changes [8]. Different molecular imaging agents for SPECT and PET had been reported in both preclinical and clinical studies [8,9,10,11,12,13,14,15,16,17]. Since IBD is an immune-mediated disease, imaging biomarkers that provide information on specific immune mediators could be more valuable in early diagnosis and evaluation of treatment response. Among potential molecular targets, integrin α4β7 is an ideal candidate for IBD imaging due to its tissue-specific role in intestinal T cell recruitment [3]. Vedolizumab (brand name: Entyvio^®^), which acts by blocking the interaction between integrin α4β7 on activated T cells and MAdCAM-1 on intestinal endothelial cells, has been approved for IBD treatment [18]. However, primary or secondary non-response to vedolizumab occurs in some patients, often due to inadequate target engagement or heterogeneous integrin α4β7 expression [19]. Integrin α4β7-targeted molecular imaging could reflect molecular-level inflammatory activity, directly confirm integrin α4β7 targeting availability before treatment, and monitor treatment response non-invasively, avoiding unnecessary therapeutic adjustments or prolonged ineffective treatment.

To fill the gap in integrin α4β7-targeted PET imaging for IBD, we developed a novel radiopharmaceutical, ^68^Ga-A2, by radiolabeling an integrin α4β7-binding peptide (DOTA-A2) with gallium-68 (^68^Ga). The targeting moiety of ^68^Ga-A2 was derived from a dimer of thioether-cyclized peptides [20]. The peptide showed relatively high α4β7 binding specificity and strong target binding (IC_50_ < 25 nM). DOTA was selected as the chelator due to its robust ^68^Ga chelation stability, compatibility with peptide conjugation, and established clinical translational potential, which meet the requirements for diagnostic imaging. A PEG6 linker was incorporated between the DOTA chelator and peptide dimer to balance synthetic feasibility, water solubility, and target accessibility. Thereafter, the DOTA conjugated peptide was afforded and denoted as DOTA-A2. This study aimed to establish ^68^Ga-A2 PET/CT imaging as a non-invasive method for detecting bowel inflammation in a DSS-induced murine colitis model. The in vitro properties (radiochemical purity, stability, binding specificity, and affinity for integrin α4β7) were validated. Then, the in vivo performance of ^68^Ga-A2 via PET/CT imaging and ex vivo biodistribution in the DSS-treated mice model was also evaluated. The results of this study may provide a new molecular imaging tool for IBD diagnosis, disease staging, and treatment follow-up.

## 2. Materials and Methods

### 2.1. Reagents and Materials

All the reagents and solvents were purchased commercially and were of analytical grade. The precursor targeting integrin α4β7 (termed DOTA-A2) was synthesized by WuXi Apptec Co., Ltd. (Wuxi, China) and the detailed synthesis procedures are shown in the Supplementary Materials. Gallium-68 (^68^Ga) was obtained from a ^68^Ge/^68^Ga generator (Eckert & Ziegler GalliaPharm, Berlin, Germany) and eluted with 0.1 mol/L hydrochloric acid. The radio high performance liquid chromatography (radio-HPLC) was conducted with an Agilent 1260 HPLC system equipped with a C_18_ column (InfinityLab Poroshell 120 EC-C18, 100 × 4.6 mml, 4 μm) (Santa Clara, CA, USA) and Flow-RAM radioactivity detector (Sheffield, UK). The integrin α4β7 protein (Catalog # IT7-H52W4) was purchased from ACROBiosystems (Beijing, China). Dextran sulfate sodium salt (molecular weight 36,000~50,000, Catalog # MB5535) was purchased from MeilunBio (Dalian, China). A Sep-Pak C_18_ Plus Light Cartridge (Cat No. WAT023501, Waters Corporation, Milford, MA, USA) was used for purification of the ^68^Ga-labeled peptide. The C_18_ cartridge was preconditioned as follows. First, 10 mL of anhydrous ethanol was used to rinse the cartridge slowly, followed by rinsing with 10 mL of ultrapure water; finally, air was gently pushed through the cartridge to remove residual water.

### 2.2. ^68^Ga Radiolabeling and Quality Control of ^68^Ga-A2

The lyophilized precursor DOTA-A2 powder was dissolved in ultrapure water to a concentration of 1 μg/μL, aliquoted into Eppendorf tubes (Protein LoBind^®^ Tubes, Cat No. 0030108116, Hamburg, Germany), and stored at −20 °C. The ^68^Ge/^68^Ga generator was eluted with 0.1 mol/L HCl, the first 1 mL of eluate was discarded, and the following 2 mL ^68^Ga eluate was collected. For every 1 mL eluate, 100 μL 1.25 mol/L sodium acetate solution was added to adjust the pH to 4.0–4.5. A total of 30 μL DOTA-A2 solution (30 μg precursor) was added to the ^68^Ga solution (about 814 MBq), mixed well, and incubated at 99 °C for 15 min. After cooling, 5 mL of ultrapure water was added and mixed. The mixture was passed through a preconditional C_18_ cartridge to remove unchelated ^68^Ga. Then, the C_18_ column was eluted with 0.5 mL 75% ethanol solution, and the eluate was diluted with 3.5 mL normal saline. Finally, the diluted solution was filtered through a 0.22 μm sterile filter membrane into a FILL-EASE^TM^ Sterile Vacuum Vial (capped) (Cat No. SVV-10C, Huayi Isotopes Co., Changshu, China) to obtain the ^68^Ga-A2 injection. Meanwhile, the radiochemical yield (RCY) was calculated as the ratio of the radioactivity of the purified ^68^Ga-labeled complex (uncorrected for decay) to the total initial radioactivity of ^68^Ga introduced into the labeling reaction, after accounting for radioactivity losses during purification, and RCY was presented as mean ± SD. For quality control, ^68^Ga-A2 injection was analyzed subsequently with radio-HPLC. Phase A was water with 0.1% TFA and phase B was ACN with 0.1% TFA. The flow rate was 1 mL/min. The gradient mobile phase started from 20% phase B and progressed to 80% phase B at 20 min.

### 2.3. Determination of the Partition Coefficient (LogD) and in Vitro Stability of ^68^Ga-A2

An aliquot of ^68^Ga-A2 (74 kBq) was added to a centrifuge tube with 5.0 mL PBS (pH 7.4) and 5.0 mL n-octanol and vortexed for 10 min. After centrifugation (20,000 rpm, 10 min), three samples (500 μL × 3) were taken from each phase and measured in a γ-counter. The cpm values were calculated as the logarithm of the octanol/PBS ratio. The determination of LogD was carried out twice with triplicate samples. The values of LogD are given as mean values ± standard deviation. For in vitro stability determination, ^68^Ga-A2 was incubated with saline for 60, 120, and 240 min and analyzed by radio-HPLC. The stability assay in 0.9% NaCl was performed in triplicate (*n* = 3).

### 2.4. In Vitro Binding Specificity and Affinity Determination

For protein binding assay, 100 μL of human integrin α4β7 (2 μg/mL) was coated onto a detachable 96-well plate at 4 °C for 24 h. The coating solution was then removed, the plates were washed three times with cold PBS, and then they were blocked with 4% BSA for 30 min. For binding specificity determination, ^68^Ga-A2 was diluted with binding buffer (20 mM Tris, 150 mM NaCl, 2 mM CaCl_2_, 1 mM MgCl_2_, 1 mM MnCl_2_, pH = 7.4) and added to the 96-well plate and incubated at 37 °C for 30 min with or without excess unlabeled A2 peptide. After incubation, the plates were washed five times with ice-cold PBS solution containing 1% BSA. These wells were then put into corresponding radioimmunoassay tubes, and the cpm of each well was measured. For binding affinity determination, ^68^Ga-A2 was diluted with binding buffer to prepare solutions of increasing concentration (0–600 nmol/L), which were added to the 96-well plate and incubated at 37 °C for 30 min. After incubation, the plates were washed five times with ice-cold PBS solution containing 1% BSA. The wells were then put into corresponding radioimmunoassay tubes, and the cpm of each well was measured, and nonlinear fitting calculations were carried out using GraphPad 10.1.2 software to obtain the apparent dissociation constant (Kd) for ^68^Ga-A2 to human integrin α4β7. Four parallel samples were set at each test point, and the experiment was repeated twice.

### 2.5. Establishment of DSS-Induced Colitis Murine Model

All animal experiments were performed in accordance with the guidelines of the Institutional Animal Care and Use Committee (IACUC) of Peking Union Medical College Hospital (approval number XHDW-2024-98, dated 24 June 2024). C57BL/6JNifdc mice (female, 6 weeks of age) were purchased from the Beijing Vital River Laboratory Animal Technology Co., Ltd. (Beijing, China) and were housed under a 12 h light/12 h dark cycle, with free access to food and water. On the basis of the published literature regarding DSS-induced colitis [21,22], and integrin α4β7-targeted imaging of IBD [23,24,25], C57BL/6JNifdc mice were treated with water containing 4% DSS (*w*/*v*) for 7 days (*n* = 20 totally) to establish the DSS-induced colitis model. For the control group, the mice were housed with normal water (*n* = 15 totally). The mice were weighed daily and assessed for clinical manifestations of colitis. The severity of the colitis was determined by a disease activity index (DAI) [26]: weight loss (0, none; 1, 1–4%; 2, 5–10%; 3, 11–20%; 4, >20%), fecal blood (0, none; 2, blood present in stool; 4, gross bleeding from the anus), and stool consistency (0, normal; 1, moist/sticky; 2, soft; 3, diarrhea; 4, bleeding). Ten mice (*n* = 5 of DSS-treated group and *n* = 5 of control group) were used for the validation and characterization of the model. On day 8, the mice were used for PET/CT imaging (*n* = 5 of DSS group and *n* = 5 of control group) and ex vivo biodistribution study (*n* = 7 of DSS group and *n* = 3 of control group). During the entire study period, if a mouse’s body weight decreases by more than 30%, the mouse would be considered to have reached a humane endpoint.

### 2.6. PET/CT Imaging and Ex Vivo Biodistribution Study in DSS-Induced Murine Colitis Model

For PET/CT imaging, ^68^Ga-A2 (100–200 μL, 7.4–11.1 MBq per mouse, 0.12–0.18 nmol, 0.41–0.62 μg) was injected into the DSS-induced murine colitis model and normal mice via tail vein. No CT contrast was administered orally or intrarectally. At 30 min post-injection, the mice were anesthetized via inhalation of 2% isoflurane and imaged using a micro-PET/CT (InliView-3000B PET/SPECT/CT, Chengdu Novel Medical Equipment Ltd., Chengdu, China). Each mouse underwent PET scanning for 15 min. The PET imaging parameters were as listed here: 511 keV photopeak and a 20% window width, with a 30 s frame duration. The parameters of the helical CT scans were 80 kVp, 0.50 mA, a 210° rotation, and a 300 ms exposure time. PET and CT images were fused using the NMSoftAIWS (NovelMedical™, Chengdu Novel Medical Equipment Ltd.). Representative PET/CT fused images were shown. For ex vivo imaging, the mice were sacrificed via cervical dislocation and the colons were removed for PET/CT imaging directly without intestinal contents removal.

For ex vivo biodistribution study, each mouse was injected with ^68^Ga-A2 (1.85 MBq per mouse, 0.03 nmol, 0.10 μg) via the tail vein and was sacrificed via cervical dislocation at 30 min post-injection. Blood, colon, and other organs of interest were harvested, weighed, and measured in a γ-counter. Blocking studies were also performed by co-injecting excess unlabeled A2 peptide (10 μg) into DSS-treated mice. Organ uptake is presented as the percentage of injected dose per gram of tissue (%ID/g).

### 2.7. Hematoxylin-Eosin (HE) Staining and Immunohistochemical (IHC) Analysis of Colon Tissues

HE staining and IHC staining of DSS-induced colitis tissue and normal colon tissue were performed as standard procedures. For integrin α4β7 staining, slides of colon tissues were incubated with a rat monoclonal antibody to mouse α4β7 (1:700; clone DATK32; BioXCell, Lebanon, NH, USA) overnight at 4 °C. Slide sections were then incubated with an HRP-conjugated goat anti-rat IgG antibody (1:200 dilution, GB23302, Servicebio, Wuhan, China) for 1 h and visualized following incubation with diaminobenzidine substrate.

### 2.8. Statistical Analysis

All statistical analyses were executed using GraphPad Prism software, version 10.1.2 (GraphPad Software Inc., San Diego, CA, USA). Data were presented as mean ± standard deviation (SD). Differences between group means were compared using Student’s *t*-test. *p* < 0.05 was considered statistically significant.

### 2.9. Utilization of GenAI Tools in This Study

During the preparation of this manuscript, the authors used Doubao AI (version 1.76.3) for specific non-core tasks (text translation and generation). All the core work (study design, experimental data collection and analysis, and result description) was performed by the authors themselves. All the outputs by GenAI were verified and reviewed strictly by authors to ensure the accuracy and reliability of the text. Text translation from Chinese to English and text generation of initial drafts of partial Introduction and Discussion based on the experimental data were performed with the assistance of Gen AI. Then, all the generated text was verified by authors (G.Y., H.Z., and L.H.). Both a cross-check with original experimental data and a validation against related references were performed and then the manuscript was revised by the authors to make the expression concise and logical.

## 3. Results

### 3.1. ^68^Ga-Radiolabeling and In Vitro Stability of ^68^Ga-A2

**Figure 1:**
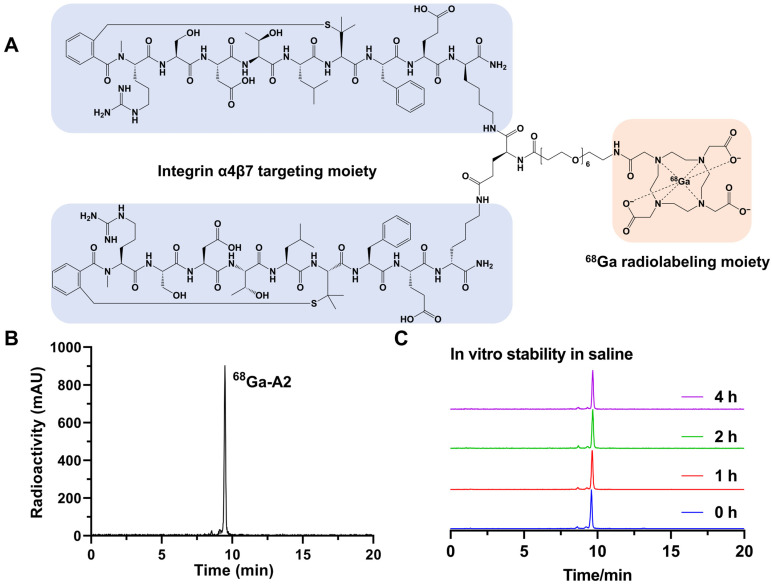
(**A**) Schematic structure of ^68^Ga-A2. Cyclic peptide targeting integrin α4β7 is marked with a blue shadow. The chelator for ^68^Ga radiolabeling is marked with an orange shadow. (**B**) Typical radio-HPLC chromatogram of ^68^Ga-A2. The radiolabeling yield of ^68^Ga-A2 was determined by radio-HPLC. (**C**) In vitro stability of ^68^Ga-A2 in saline for different times. The peak sizes were normalized with a consistent Y-axis range.

The labeling precursor (A2) was prepared successfully with high chemical purity (>95%) and identified by mass spectrometry with a calculated molecular weight of 3343.82 Da. The detailed synthesis procedures and results are shown in the Supplementary Materials. ^68^Ga-A2 was prepared by reacting ^68^Ga^3+^ with DOTA-A2 in the condition of pH = 4.0. Radiolabeling was completed by heating the reaction mixture at 99 °C for 15 min. Schematic structures of ^68^Ga-A2 were shown in Figure 1A. ^68^Ga-A2 consisted of integrin α4β7-targeting peptides and a ^68^Ga-chelating moiety, which were connected via a linker in the middle. ^68^Ga-A2 was afforded with a radiochemical purity >95% (Figure 1B), a radiochemical yield of 66.01 ± 4.00% (uncorrected for decay), and a specific activity of 60.83 MBq/nmol. Although ^68^Ga-A2 could maintain high stability in saline with no obvious decomposition peaks observed even after 4 h (Figure 1C), distinct degradation was observed during serum incubation and urine sample analysis, indicating suboptimal in vivo stability of ^68^Ga-A2 (Figure S5). In addition, the n-Octanol/PBS distribution coefficient (LogD) value of ^68^Ga-A2 was determined to be −1.90 ± 0.16, suggesting that ^68^Ga-A2 was hydrophilic.

### 3.2. In Vitro Characterization of ^68^Ga-A2

**Figure 2:**
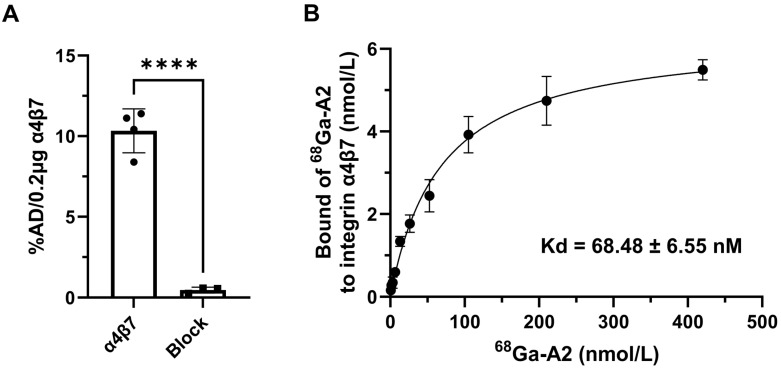
In vitro characterization of ^68^Ga-A2. (**A**) Binding of ^68^Ga-A2 to human integrin α4β7 protein with and without blocking of unlabeled A2 peptide (*n* = 4). %AD/0.2 μg protein = percentage of total added dose per 0.2 μg human integrin α4β7 protein. **** means *p* value < 0.001. (**B**) The dissociation constant (Kd) of ^68^Ga-A2 to human integrin α4β7 protein was determined by saturation binding experiment (*n* = 4). The saturation curve is shown with a Kd of 68.48 ± 6.55 nM.

The binding specificity and affinity of ^68^Ga-A2 to human integrin α4β7 were determined by protein binding assay. ^68^Ga-A2 exhibited significant binding values (presented as %AD per 0.2 μg protein) for integrin α4β7 (10.33 ± 1.36). The specific binding was significantly inhibited by treating with excess unlabeled A2 peptide (0.46 ± 0.18, *p* < 0.0001) (Figure 2A). The dissociation constant Kd of ^68^Ga-A2 to integrin α4β7 was 68.48 ± 6.55 nM, which showed relatively high affinity among peptide-based radiopharmaceuticals.

### 3.3. Induction and Confirmation of DSS-Induced Colitis

**Figure 3:**
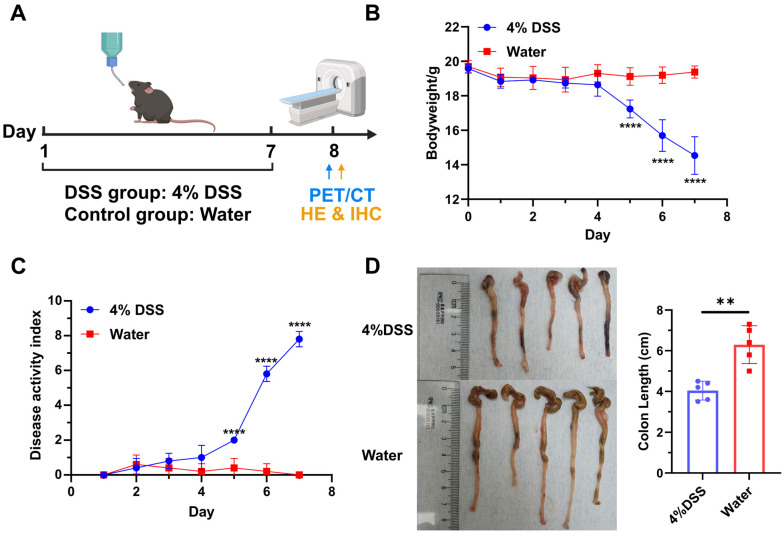
Establishment and evaluation of DSS-induced murine colitis model (*n* = 5). (**A**) Schematic illustration of model establishment and study design. Created in BioRender. Yang, G. (2025) https://BioRender.com/o9tmwhh. (**B**) Bodyweight change in mice treated with 4% DSS or normal water. (**C**) Disease activity index (DAI) scores of mice treated with 4% DSS or normal water. (**D**) Comparison of colon length between control group and DSS-treated group. **** means *p* value < 0.001, ** means *p* value < 0.01. “4% DSS” and “Water” in the subfigures indicate the group treated with 4% DSS and normal water, respectively.

Colitis was induced in C57BL/6J mice by providing 4% DSS in water ad libitum for 7 days, followed by recovery with regular drinking water on day 8 (Figure 3A). Meanwhile, the mice were used for the PET/CT imaging or biodistribution study on day 8. The colon tissues were further used for HE staining and integrin α4β7 IHC staining. DSS-treated mice showed weight loss on day 5 (Figure 3B). On day 7, the weight of DSS-treated mice was significantly reduced to 74% ± 0.04% of initial weight, whereas control mice did not display any weight loss (*p* < 0.0001; *n* = 5). In addition, from day 5, DSS-treated mice also had significantly higher disease activity index scores than control mice as a result of increased weight loss, soft stool/diarrhea, and fecal bleeding (Figure 3C). The colons were also removed and their lengths were measured. The colons of DSS-treated mice were collectively shorter than those of healthy mice, with the values of 4.04 ± 0.46 cm and 6.30 ± 0.93 cm (*p* = 0.0012, *n* = 5), respectively (Figure 3D). The above results indicated that the DSS-induced colitis model was successfully established.

### 3.4. PET/CT Imaging of ^68^Ga-A2 in DSS-Induced Colitis Model

**Figure 4:**
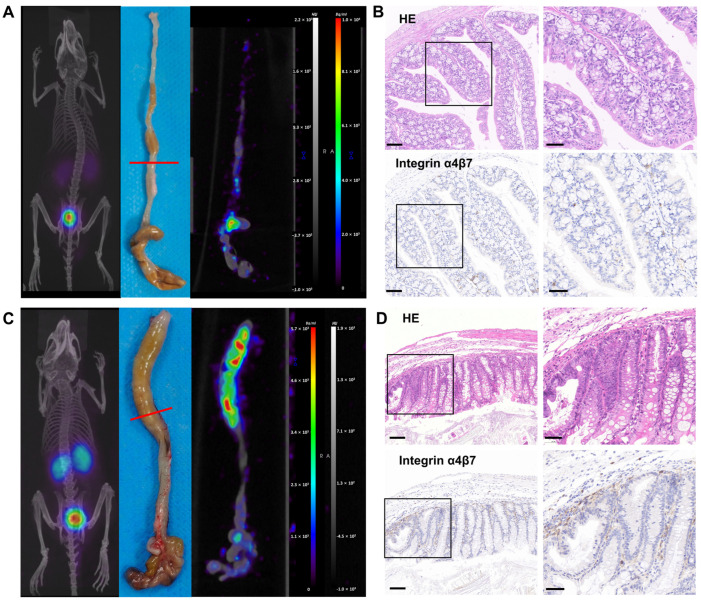
(**A**) ^68^Ga-A2 PET/CT imaging in healthy mice at 30 min post-injection. Representative PET/CT fused image and PET image of colon were shown. The red line indicated the location of colon tissue for HE and integrin α4β7 IHC staining. (**B**) HE and integrin α4β7 IHC staining of colon tissue in control group. Scale bars: left panel, 100 μm; right panel, 50 μm. (**C**) ^68^Ga-A2 PET/CT imaging in DSS-treated mice at 30 min post-injection. Representative PET/CT fused image and PET image of colon are shown. The red line indicated the location of colon tissue for HE and integrin α4β7 IHC staining. (**D**) HE and integrin α4β7 IHC staining of colon tissue in DSS-treated group. Scale bars: left panel, 100 μm; right panel, 50 μm.

To evaluate the in *vivo* pharmacokinetics of ^68^Ga-A2, micro-PET/CT imaging in both the DSS-induced colitis model and healthy mice was performed on day 8 (Figure 3A and Figure 4). ^68^Ga-A2 (11.1 MBq, 100 μL) was injected into each mouse and imaged at 30 min post-injection (*n* = 5). ^68^Ga-A2 was excreted mainly via the kidneys and urinary system rapidly, resulting in high radioactive signal intensity in the kidneys and bladder (Figure 4A). On maximum intensity projection (MIP) images, no visible radioactive signals in colon tissues were observed in the DSS-induced colitis mice by visual assessment, which was indistinguishable from the control group (Figure 4C). Subsequently, the mice were sacrificed, and the colonic tissues were harvested for ex vivo imaging. It was found that ^68^Ga-A2 showed focal uptake in the intestinal tissues of DSS-induced mice, with significantly higher radioactive signal intensity than that in the control group by visual evaluation (Figure 4C, Supplementary Figure S3). It was found that ^68^Ga-A2 showed focal uptake in the intestinal tissues of DSS-treated mice, with significantly higher radioactive signal intensity than that in the control group by visual evaluation (Figure 4C and Figure S3). Histological analysis of the intestinal tissues with HE staining and integrin α4β7 IHC staining revealed obvious mucosal destruction and infiltration of integrin α4β7-positive cells in the intestines of DSS-treated mice (Figure 4D). In contrast, the intestinal mucosal epithelium of control mice was structurally intact, and the number of integrin-positive cells was significantly lower than that in the DSS group (Figure 4B). These findings confirmed the underlying reason for the differential uptake of ^68^Ga-A2 in intestinal tissues at the histological level. However, ^68^Ga-A2 failed to detect colitis lesions on MIP images. This may be attributed to the scattered infiltration of integrin α4β7-expressing T cells into the damaged submucosa in inflammatory bowel disease, leading to diffuse uptake of ^68^Ga-A2, which might be filtered out during image reconstruction. On the other hand, the affinity of A2 for integrins was merely 68.48 ± 6.55 nM, which was weaker than that of antibody-based radioprobes, resulting in suboptimal imaging performance on MIP images.

### 3.5. Ex Vivo Biodistribution Study of ^68^Ga-A2 in DSS-Induced Colitis Model

**Figure 5:**
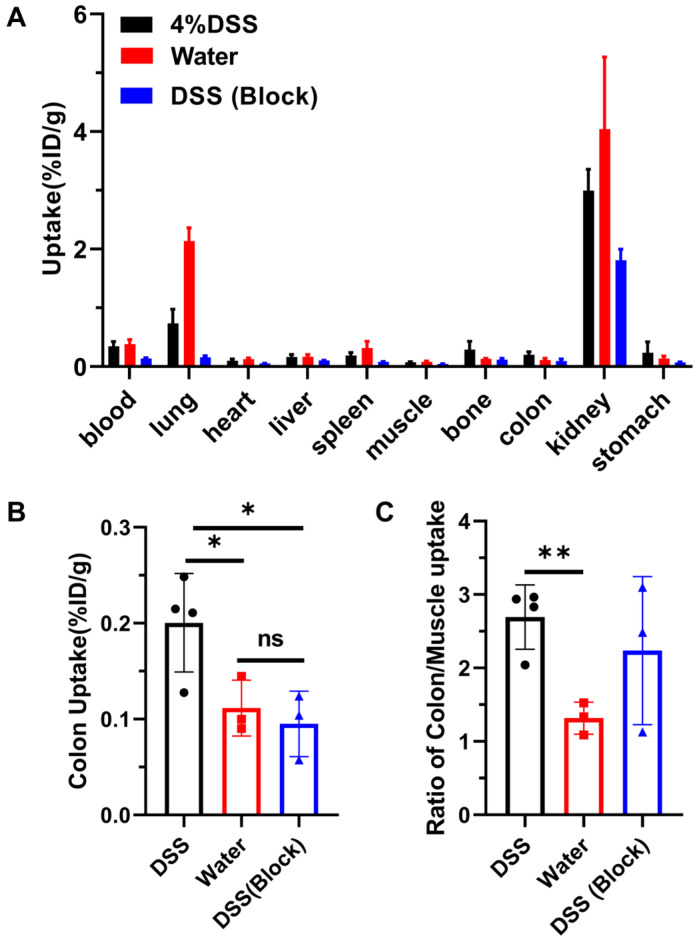
(**A**) Ex vivo biodistribution of various tissues and organs in control group (*n* = 3) and DSS groups (*n* = 4 for DSS group and *n* = 3 for blocking group) at 30 min post-injection. The mice in block group were co-injected with ^68^Ga-A2 and unlabeled A2 peptide. (**B**,**C**) Colon uptake and ratios of colon to muscle uptake were analyzed. ** means *p* value < 0.01, * means *p* value < 0.05.

Biodistribution of ^68^Ga-A2 in DSS-induced colitis model at 30 min post-injection was conducted. We found that ^68^Ga-A2 was excreted quickly from kidney, leaving very low uptake in other organs except for the lungs (Figure 5A, Table 1). The uptake value of ^68^Ga-A2 in the colon (0.20 ± 0.05%ID/g) was significantly higher than that in healthy mice (0.11 ± 0.03%ID/g, *p* = 0.0450) and the blocking group (0.10 ± 0.03%ID/g, *p* = 0.0283) (Figure 5B). Meanwhile, it was observed that even within the DSS-induced colitis group, there was heterogeneity in ^68^Ga-A2 uptake among different mice, with values ranging from 0.13 to 0.25%ID/g. Further analysis showed that the colon/muscle ratio in the DSS group (2.69 ± 0.44) was significantly higher than that in the healthy group (1.32 ± 0.22, *p* = 0.0044), but no significant difference compared with the blocking group (2.24 ± 1.01, *p* = 0.4454) was observed (Figure 5C). Notably, the uptake in the lungs of mice in control group was significantly higher than other groups (Figure 5A, Table 1), but the underlying biological mechanism remained unknown and may require further investigation. In addition, we also observed a significant decrease in accumulation of ^68^Ga-A2 in the non-target tissues of the blocked versus unblocked DSS cohorts, such as blood, heart, spleen, muscle, kidneys, and stomach (Figure 5A, Table 1). A rationale for this is that excess unlabeled A2 peptide might accelerate the excretion or reduce the renal reabsorption of ^68^Ga-A2, resulting in lower tracer binding in the whole body, but further studies should be performed to explore the underlying biological mechanisms.

## 4. Discussion

This study successfully prepared ^68^Ga-A2, a radiopharmaceutical targeting integrin α4β7. Radiolabeling was achieved quickly and conveniently. No obvious decomposition was observed within 4 h in saline, and this stability feature laid a crucial foundation for its further application (Figure 1). Meanwhile, ^68^Ga-A2 has a LogD value of −1.90 ± 0.16, indicating strong hydrophilicity. Hydrophilic molecules are usually excreted through the kidneys, which is highly consistent with the high kidney–bladder uptake phenomenon observed in subsequent in vivo PET imaging. This further verifies the reasonable clearance pathway of ^68^Ga-A2, which can reduce radioactive accumulation in non-target organs and lower potential radiation risks. ^68^Ga-A2 exhibits specific binding ability to human integrin α4β7. In addition, the dissociation constant Kd of ^68^Ga-A2 with integrin α4β7 is 68.48 ± 6.55 nM (Figure 2). Although its affinity is lower than that of antibody-based radiopharmaceuticals (usually in the pM-nM range), it still belongs to a high-affinity level among peptide-based radiopharmaceuticals. Compared with antibodies, peptide probes have advantages such as small molecular weight, strong tissue penetration, and rapid in vivo clearance [27]. The affinity characteristics of ^68^Ga-A2 enabled it to effectively bind to target molecules while retaining the advantages of peptide probes, providing a molecular basis for subsequent in vivo imaging.

In this study, the well-established DSS-induced colitis model of IBD was selected for its high similarity to human UC (e.g., mucosal-dominant inflammation, continuous lesions) and favorable reproducibility [28,29]. The bodyweight changes, disease activity index scores, colon length, and HE staining of DSS-treated mice collectively confirmed the successful establishment of the DSS-induced colitis model (Figure 3 and Figure 4D). Subsequently, we performed PET/CT imaging and biodistribution studies of ^68^Ga-A2 in DSS-induced mice and healthy mice. Notably, ^68^Ga-A2 showed contradictory results of negative whole-body imaging and positive ex vivo imaging. In the MIP images at 30 min post-injection, no obvious radioactive uptake was observed visually in the colonic inflammatory foci of DSS-treated mice, which showed no significant difference from the control group. However, ex vivo imaging of the colon showed that the radioactive signal intensity of colon tissue in the DSS group was significantly higher than that in the control group based on visual evaluation (Figure 4 and Figure S3), and this result was consistent with the results of HE staining and integrin α4β7 IHC staining. The potential reasons for this contradiction may include the following three aspects. Firstly, under the pathological state of IBD, integrin α4β7-positive T cells in inflammatory foci are scattered in the damaged submucosa, leading to diffuse distribution of ^68^Ga-A2 uptake in inflammatory foci, which could be confirmed by IHC. However, the spatial resolution of PET/CT imaging is limited (the spatial resolution of micro-PET used in this study is about 1.1 mm), and the radioactive signal of diffuse uptake may be filtered during image reconstruction, making it impossible to be identified in whole-body imaging. Secondly, although ^68^Ga-A2 has relatively high affinity among peptide-based drugs (Kd = 68.48 nM), its binding strength to integrin α4β7 is still lower than that of antibody-based radiopharmaceuticals (such as antibodies targeting integrin α4β7 [25] or TNF-α [30,31]), which may lead to insufficient accumulation of radioactive signals in inflammatory foci, failing to break through background noise in whole-body imaging and thus being difficult to detect. Thirdly, the in vivo clearance pathway of ^68^Ga-A2 may also affect the imaging results. Since ^68^Ga-A2 is mainly excreted through the kidneys, the radioactive signal intensity of the kidneys and bladder is high 30 min post-injection, which may interfere with the imaging of the abdominal colon region. Especially in the case of small mouse size and dense distribution of abdominal organs, the high background signal further masks the weak radioactive signal of inflammatory foci, resulting in the inability to observe specific uptake of inflammatory foci in whole-body imaging. In addition, the rapid renal clearance of ^68^Ga-A2 might also limit its target binding efficacy. In this study, intestinal contents were not removed during ex vivo colonic processing to avoid disrupting the integrity of inflamed mucosal tissue and introducing irreproducible artifacts, with strict consistency in tissue handling across all groups ensuring uniform potential influence of intestinal contents. This approach aligns with established protocols in preclinical IBD imaging research, as demonstrated in recent studies where ex vivo colonic imaging without content removal was successfully used to validate tracer targeting specificity [9,26]. Nevertheless, ex vivo imaging and biodistribution studies both prove that ^68^Ga-A2 can specifically target colonic inflammatory foci in vivo, echoing the results of in vitro binding experiments and histopathology.

Current clinical imaging technologies for IBD mainly include endoscopy, CT/MRI enterography, and ^18^F-FDG PET/CT (or PET/MRI) [32,33,34]. Endoscopy is invasive and unable to evaluate deep intestinal wall inflammation and extraintestinal lesions. CT/MRI rely on structural changes in the intestine and lack molecular-level inflammation assessment or sufficient temporal resolution for disease monitoring. ^18^F-FDG PET/CT offers non-invasive whole-body imaging with high sensitivity but lacks tissue specificity, leading to false-positive results [35]. Especially in patients with concurrent tumors or colitis-associated colorectal cancer (CAC), the diagnostic accuracy is limited [36]. To date, few integrin α4β7-targeted radiotracers labeled with ^64^Cu or ^99m^Tc were also reported for IBD imaging [24,25]. These antibody-based radiotracers showed high hepatic uptake, long circulation times, and needed extra-long time for optimal imaging contrast, which reduced imaging sensitivity and the feasibility of clinical application. Herein, ^68^Ga-A2 showed specific binding to integrin α4β7 and exhibited advantages for molecular-level targeting of intestinal inflammation, as supported by our preclinical in vitro binding assays and in vivo biodistribution data. This differentiates it from structural imaging or non-specific metabolic tracers, laying the foundation for functional imaging. And ^68^Ga-A2 undergoes rapid post-injection distribution, allowing visualization of colitis lesions as early as 30 min post-injection. Although ^68^Ga-A2 did not detect inflammatory foci in whole-body PET/CT imaging in this study, ex vivo imaging and biodistribution results have confirmed its targeting ability. Through further optimization of drug structure (such as improving binding affinity and in vivo stability through D-amino acids or unnatural amino acids substitution [37], optimizing the linker to balance hydrophilicity and clearance rate, and prolonging the blood half-life of the probe by introducing an albumin-binding moiety [38,39]) and adjustment of imaging parameters (such as optimizing image reconstruction algorithms), its imaging effect is expected to be improved, making it more suitable for non-invasive imaging of IBD inflammatory foci. Additionally, further studies are also required before validating its utility in patient populations, which will be the focus of our subsequent work (e.g., optimizing the molecular structure, conducting large-animal validation, toxicity studies, GMP (Good Manufacturing Practice)-compliant production, dosimetry determination, and designing clinical pilot studies).

This study has several limitations. First, the animal model has limitations. Although the DSS-induced colitis model has a high similarity to human UC, it still cannot fully simulate the complexity of human IBD. For example, the pathogenesis of human IBD is related to multiple factors such as genetics, environment, and gut microbiota, while the DSS model mainly induces inflammation through chemical damage, lacking the participation of these complex factors [2,28,40]. Therefore, the research results need to be carefully verified in other mouse models of IBD. In addition, we conducted animal experiments exclusively in female mice, which may have overlooked the impact of gender differences in the colitis mouse model [41], thereby limiting the broad applicability of the study’s conclusions. Second, insufficient drug optimization. The affinity and in vivo stability of ^68^Ga-A2 still have room for improvement. Further optimization of the drug’s molecular structure is needed to improve the efficiency and specificity of targeted binding. Finally, no treatment response monitoring or prediction of therapeutic efficacy study was conducted. This study only evaluated the imaging effect of ^68^Ga-A2 in the acute phase of inflammation and did not explore its changes after IBD treatment (such as vedolizumab). Future research should aim to optimize DOTA-A2 to improve the binding affinity and specificity of ^68^Ga-A2 to integrin α4β7, and conduct treatment response monitoring studies to evaluate its value in efficacy evaluation and provide a basis for clinical treatment decisions. Additionally, the mesenteric lymph nodes (MLNs) serve as critical hubs for immune cell activation and homing to the inflamed colon in IBD models. However, MLNs were not included as a focus of our current study. Further investigations should be performed to systematically evaluate radiotracer uptake in MLNs and correlation between the uptake with integrin α4β7 expression.

## 5. Conclusions

^68^Ga-A2, a ^68^Ga-labeled integrin α4β7-targeted radiopharmaceutical with favorable characteristics, has been verified in vitro and in the DSS-induced murine colitis model. Despite limitations in whole-body imaging, ^68^Ga-A2 is expected to be a promising non-invasive PET imaging agent for detecting inflammatory foci in IBD through subsequent optimization and refinement. These findings warrant further optimization of ^68^Ga-A2 and clinical investigations to validate its feasibility and utility in IBD patients.

## Figures and Tables

**Table 1:** Biodistribution of tissues and organs in control group and DSS modeling group (%ID/g).

Tissue	Group 1 DSS (*n* = 4)	Group 2 Water (*n* = 3)	Group 3 DSS (Block) (*n* = 3)	*p* Value		
				1 vs. 3	1 vs. 2	2 vs. 3
Blood	0.34 ± 0.08	0.38 ± 0.08	0.14 ± 0.02	0.0081	0.5512	0.0057
Lung	0.74 ± 0.24	2.14 ± 0.23	0.16 ± 0.03	0.0095	0.0005	0.0001
Heart	0.10 ± 0.03	0.13 ± 0.02	0.05 ± 0.00	0.0303	0.2356	0.0033
Liver	0.16 ± 0.04	0.17 ± 0.04	0.11 ± 0.01	0.0602	0.8765	0.0372
Spleen	0.19 ± 0.05	0.32 ± 0.11	0.08 ± 0.01	0.0114	0.0925	0.0220
Muscle	0.07 ± 0.01	0.08 ± 0.01	0.04 ± 0.01	0.0074	0.2409	0.0041
Bone	0.29 ± 0.14	0.13 ± 0.01	0.12 ± 0.03	0.0910	0.1109	0.4249
Colon	0.20 ± 0.05	0.11 ± 0.03	0.10 ± 0.03	0.0283	0.0450	0.5581
Kidney	2.99 ± 0.36	4.04 ± 1.23	1.81 ± 0.18	0.0039	0.1581	0.0359
Stomach	0.24 ± 0.19	0.14 ± 0.04	0.07 ± 0.01	0.2052	0.4260	0.0624

## Data Availability

The original contributions presented in this study are included in the article/Supplementary Materials. Further inquiries can be directed to the corresponding author.

## Supplementary Materials

The following supporting information can be downloaded at https://www.mdpi.com/article/10.3390/pharmaceutics17121591/s1, Figure S1. Synthetic scheme of A2; Figure S2. Representative HPLC chromatogram images and TOF MS results of A2; Figure S3. Ex vivo PET imaging of colons of DSS-induced mice and healthy mice; Figure S4. Acute toxicity study of ^68^Ga-A2 in normal mice. Figure S5. In vitro stability (in mouse serum) and in vivo stability (urine samples) of ^68^Ga-A2; Table S1. The list of amino acids and the corresponding reagents used on SPPS during the synthesis of DOTA-A2.

## References

[1] Vieujean S., Jairath V., Peyrin-Biroulet L., Dubinsky M., Iacucci M., Magro F., Danese S. (2025). Understanding the Therapeutic Toolkit for Inflammatory Bowel Disease. Nat. Rev. Gastroenterol. Hepatol..

[2] Ramos G.P., Papadakis K.A. (2019). Mechanisms of Disease: Inflammatory Bowel Diseases. Mayo Clin. Proc..

[3] Lamb C.A., O’Byrne S., Keir M.E., Butcher E.C. (2018). Gut-Selective Integrin-Targeted Therapies for Inflammatory Bowel Disease. J. Crohns Colitis.

[4] Hong S.M., Baek D.H. (2024). Diagnostic Procedures for Inflammatory Bowel Disease: Laboratory, Endoscopy, Pathology, Imaging, and Beyond. Diagnostics.

[5] Nett A., Velayos F., McQuaid K. (2014). Quality Bowel Preparation for Surveillance Colonoscopy in Patients with Inflammatory Bowel Disease Is a Must. Gastrointest. Endosc. Clin. N. Am..

[6] Molinié F., Gower-Rousseau C., Yzet T., Merle V., Grandbastien B., Marti R., Lerebours E., Dupas J.-L., Colombel J.-F., Salomez J.-L. (2004). Opposite Evolution in Incidence of Crohn’s Disease and Ulcerative Colitis in Northern France (1988–1999). Gut.

[7] Dmochowska N., Wardill H.R., Hughes P.A. (2018). Advances in Imaging Specific Mediators of Inflammatory Bowel Disease. Int. J. Mol. Sci..

[8] Rezazadeh F., Kilcline A.P., Viola N.T. (2023). Imaging Agents for PET of Inflammatory Bowel Disease: A Review. J. Nucl. Med..

[9] Salehi Farid A., Rowley J.E., Allen H.H., Kruger I.G., Tavakolpour S., Neeley K., Cong M., Shahbazian H., Dorafshani N., Berrada A. (2025). CD45-PET Is a Robust, Non-Invasive Tool for Imaging Inflammation. Nature.

[10] Zhou W., Ran J., Hu X., Lv C., You J., Sun D., Chen L., Tang Y., Li H., Hu D. (2025). 18F-FAPI PET/CT for Early Detection and Severity Assessment of Intestinal Fibrosis in a Mouse Model. Inflamm. Bowel Dis..

[11] Pan Q., Xu H., Liu S., Zhang H., Zhang S., Li J., Li F., Luo Y. (2025). Head-to-Head Comparison of 68Ga-FAPI-04 and 18F-FDG PET/CT for the Assessment of Crohn’s Disease: A Prospective Pilot Study. Clin. Nucl. Med..

[12] Li X., Liu Y., Zhang Z., Hai W., Pan Y., Zhang Y. (2025). Exendin-4 Imaging Based on Gastrointestinal GLP-1R Targets for IBD Diagnosis and Efficacy Assessment. Eur. J. Nucl. Med. Mol. Imaging.

[13] Heidari P., Haj-Mirzaian A., Prabhu S., Ataeinia B., Esfahani S.A., Mahmood U. (2024). Granzyme B PET Imaging for Assessment of Disease Activity in Inflammatory Bowel Disease. J. Nucl. Med..

[14] Chen Y., Yuan H., Tan X., Shang Y., Sun X., Wang P., Jiang L. (2024). CXCR4-Targeted 68 Ga-Pentixafor PET/CT Imaging in Inflammatory Bowel Disease. Clin. Nucl. Med..

[15] Bhowmik A.A., Heikkilä T.R.H., Polari L., Virta J., Liljenbäck H., Moisio O., Li X.-G., Viitanen R., Jalkanen S., Koffert J. (2024). Detection of Intestinal Inflammation by Vascular Adhesion Protein-1-Targeted [68Ga]Ga-DOTA-Siglec-9 Positron Emission Tomography in Murine Models of Inflammatory Bowel Disease. Mol. Imaging Biol..

[16] Ismail M.S., Peters D.E., Rowe S.P., Salavati A., Sharma S., Anders R.A., Pomper M., Slusher B.S., Selaru F.M. (2023). PSMA-Targeted PET Radiotracer [18F]DCFPyL as an Imaging Biomarker in Inflammatory Bowel Disease. Clin. Exp. Gastroenterol..

[17] Aarntzen E.H.J.G., Hermsen R., Drenth J.P.H., Boerman O.C., Oyen W.J.G. (2016). 99mTc-CXCL8 SPECT to Monitor Disease Activity in Inflammatory Bowel Disease. J. Nucl. Med..

[18] Jovani M., Danese S. (2013). Vedolizumab for the Treatment of IBD: A Selective Therapeutic Approach Targeting Pathogenic A4b7 Cells. Curr. Drug Targets.

[19] Peyrin-Biroulet L., Danese S., Argollo M., Pouillon L., Peppas S., Gonzalez-Lorenzo M., Lytras T., Bonovas S. (2019). Loss of Response to Vedolizumab and Ability of Dose Intensification to Restore Response in Patients with Crohn’s Disease or Ulcerative Colitis: A Systematic Review and Meta-Analysis. Clin. Gastroenterol. Hepatol. Off. Clin. Pract. J. Am. Gastroenterol. Assoc..

[20] Bhandari A., Patel D.V., Zemede G., Frederick B.T., Mattheakis L.C. (2017). α_4_β_7_ Integrin Thioether Peptide Antagonists. U.S. Patent.

[21] Hall L.J., Faivre E., Quinlan A., Shanahan F., Nally K., Melgar S. (2011). Induction and Activation of Adaptive Immune Populations During Acute and Chronic Phases of a Murine Model of Experimental Colitis. Dig. Dis. Sci..

[22] Bonfiglio R., Galli F., Varani M., Scimeca M., Borri F., Fazi S., Cicconi R., Mattei M., Campagna G., Schönberger T. (2021). Extensive Histopathological Characterization of Inflamed Bowel in the Dextran Sulfate Sodium Mouse Model with Emphasis on Clinically Relevant Biomarkers and Targets for Drug Development. Int. J. Mol. Sci..

[23] Dearling J.L.J., Park E.J., Dunning P., Baker A., Fahey F., Treves S.T., Soriano S.G., Shimaoka M., Packard A.B., Peer D. (2010). Detection of Intestinal Inflammation by MicroPET Imaging Using a 64Cu-Labeled Anti-Beta7 Integrin Antibody. Inflamm. Bowel Dis..

[24] Dearling J.L.J., Daka A., Veiga N., Peer D., Packard A.B. (2016). Colitis ImmunoPET: Defining Target Cell Populations and Optimizing Pharmacokinetics. Inflamm. Bowel Dis..

[25] Signore A., Bonfiglio R., Varani M., Galli F., Campagna G., Desco M., Cussó L., Mattei M., Wunder A., Borri F. (2023). Radioimmune Imaging of α_4_β_7_ Integrin and TNFα for Diagnostic and Therapeutic Applications in Inflammatory Bowel Disease. Pharmaceutics.

[26] Freise A.C., Zettlitz K.A., Salazar F.B., Tavaré R., Tsai W.-T.K., Chatziioannou A.F., Rozengurt N., Braun J., Wu A.M. (2018). Immuno-PET in Inflammatory Bowel Disease: Imaging CD4-Positive T Cells in a Murine Model of Colitis. J. Nucl. Med..

[27] Wynendaele E., Bracke N., Stalmans S., De Spiegeleer B. (2014). Development of Peptide and Protein Based Radiopharmaceuticals. Curr. Pharm. Des..

[28] Yang C., Merlin D. (2024). Unveiling Colitis: A Journey through the Dextran Sodium Sulfate-Induced Model. Inflamm. Bowel Dis..

[29] Chassaing B., Aitken J.D., Malleshappa M., Vijay-Kumar M. (2014). Dextran Sulfate Sodium (DSS)-Induced Colitis in Mice. Curr. Protoc. Immunol..

[30] Codesido J., García-Varela L., García-Otero X., Bouzón-Barreiro S., Gómez-Lado N., Toja-Camba F.J., Mondelo-García C., Lazaré H., Torres J.B., Vidal-Otero J. (2025). PET Biodistribution Study of Subcutaneous and Intravenous Administration of Adalimumab in an Inflammatory Bowel Disease Model. Int. J. Pharm..

[31] Yan G., Wang X., Fan Y., Lin J., Yan J., Wang L., Pan D., Xu Y., Yang M. (2022). Immuno-PET Imaging of TNF-α in Colitis Using 89Zr-DFO-Infliximab. Mol. Pharm..

[32] Kumar R., Melmed G.Y., Gu P. (2024). Imaging in Inflammatory Bowel Disease. Rheum. Dis. Clin. N. Am..

[33] Borhani A., Afyouni S., Attari M.M.A., Mohseni A., Catalano O., Kamel I.R. (2023). PET/MR Enterography in Inflammatory Bowel Disease: A Review of Applications and Technical Considerations. Eur. J. Radiol..

[34] Shaban N., Hoad C.L., Naim I., Alshammari M., Radford S.J., Clarke C., Marciani L., Moran G. (2022). Imaging in Inflammatory Bowel Disease: Current and Future Perspectives. Frontline Gastroenterol..

[35] Brodersen J.B., Hess S. (2020). FDG-PET/CT in Inflammatory Bowel Disease: Is There a Future?. PET Clin..

[36] Shah S.C., Itzkowitz S.H. (2022). Colorectal Cancer in Inflammatory Bowel Disease: Mechanisms and Management. Gastroenterology.

[37] Di L. (2015). Strategic Approaches to Optimizing Peptide ADME Properties. AAPS J..

[38] Lau J., Jacobson O., Niu G., Lin K.-S., Bénard F., Chen X. (2019). Bench to Bedside: Albumin Binders for Improved Cancer Radioligand Therapies. Bioconjug. Chem..

[39] Davis R.A., Hausner S.H., Harris R., Sutcliffe J.L. (2022). A Comparison of Evans Blue and 4-(p-Iodophenyl)Butyryl Albumin Binding Moieties on an Integrin α_v_β_6_ Binding Peptide. Pharmaceutics.

[40] Lu Q., Yang M.-F., Liang Y.-J., Xu J., Xu H.-M., Nie Y.-Q., Wang L.-S., Yao J., Li D.-F. (2022). Immunology of Inflammatory Bowel Disease: Molecular Mechanisms and Therapeutics. J. Inflamm. Res..

[41] Bábíčková J., Tóthová Ľ., Lengyelová E., Bartoňová A., Hodosy J., Gardlík R., Celec P. (2015). Sex Differences in Experimentally Induced Colitis in Mice: A Role for Estrogens. Inflammation.

