# Supplementary material for: A Novel 68Ga-Labeled Integrin α4β7-Targeted Radiopharmaceutical for PET/CT Imaging of DSS-Induced Murine Colitis
Source: Pharmaceutics. 2025 Dec 10;17(12):1591. doi: 10.3390/pharmaceutics17121591 (PMC12736890; doi:10.3390/pharmaceutics17121591)
Supplement: Supplementary file 1 [file pharmaceutics-17-01591-s001.zip › pharmaceutics-3990761-supplementary.pdf]

Supplementary information for Article

A Novel ^{68}Ga-Labeled Integrin α4β7-Targeted Radiopharmaceutical for PET/CT Imaging of DSS-Induced Murine Colitis

Guangjie Yang, Haiqiong Zhang and Li Huo *

Department of Nuclear Medicine, State Key Laboratory of Complex, Severe, and Rare Diseases, Center for Rare Diseases Research, Peking Union Medical College Hospital, Chinese Academy of Medical Science and Peking Union Medical College, Beijing 100730, China; yangguangjie@pumch.cn (G.Y.); hqzhang22@163.com (H.Z.)

* Correspondence: huoli@pumch.cn

1. Synthesis of A2

The synthetic route is shown in Figure S1.

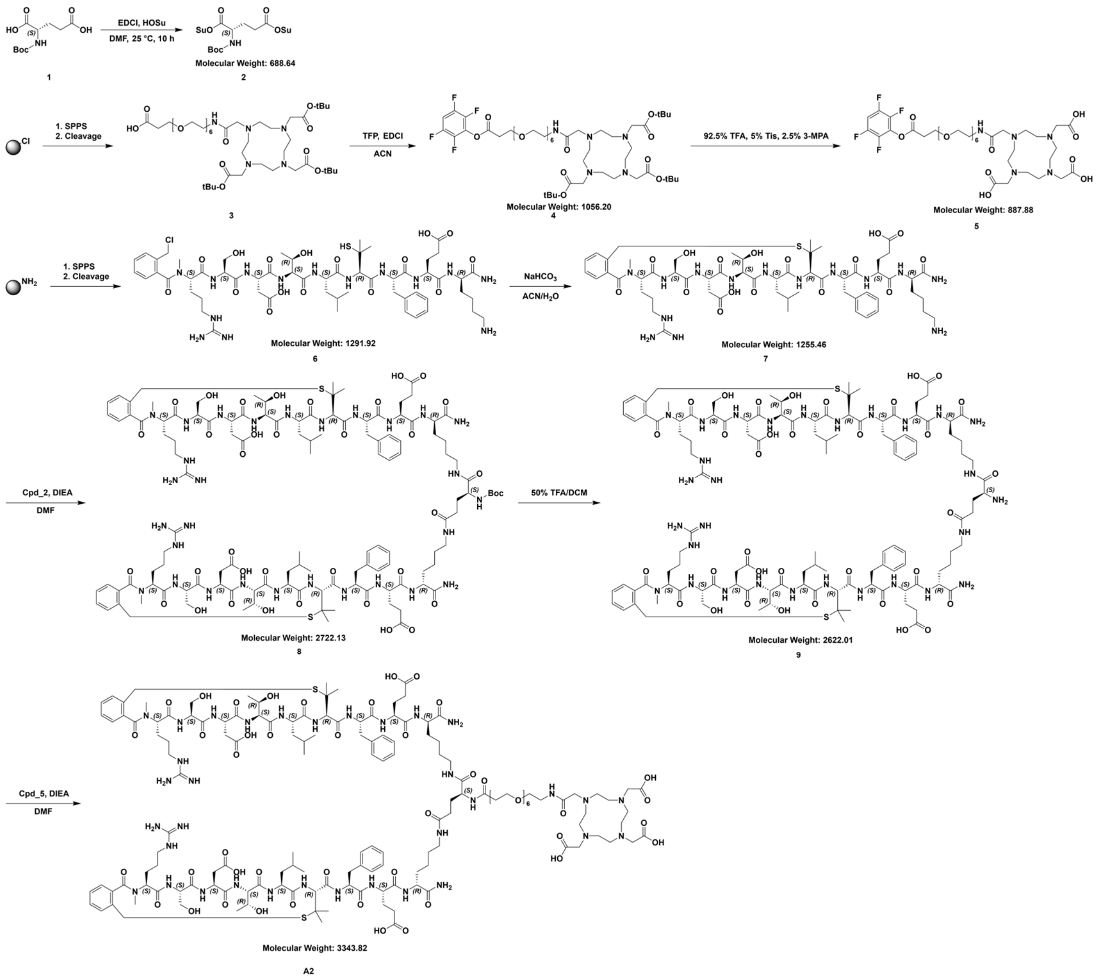

Figure S1. Synthetic Scheme of A2.

(1) **Synthesis of compound 2**.

To a solution of compound **1** (1.50 g, 6.07 mmol, 1.00 equiv.) and HOSu (1.75 g, 15.1 mmol, 2.50 equiv.) in DMF (15.0 mL) was added EDCI (2.91 g, 15.1 mmol, 2.50 equiv.) and stirred 10 h at

25 °C. LCMS showed the compound **1** was consumed and one peak (R_t = 0.36 min) with desired mass was detected. H_2O (15.0 mL) was added to the reaction mixture. Then the mixture was extracted with EtOAc (15.0 mL * 2). The combined organic phase was washed with 10% citric acid (30.0 mL * 3), sat. $NaHCO_3$ solution (30.0 mL * 3), H_2O (40.0 mL) and brine (30.0 mL), dried over Na_2SO_4, concentrated under reduced pressure to give compound **2** (2.50 g, 5.66 mmol, 93.3% yield) as a white solid, confirmed by HNMR.

(2) **Synthesis of compound 3**.

Peptide Synthesis: the peptide was synthesized using standard Fmoc chemistry.

1) Resin preparation: To the vessel containing 2-CTC Resin (2.00 mmol, 1.82 g, Sub = 1.10 mmol/g), Fmoc-NH-PEG6-CH_2CH_2COOH (1.15 g, 2.00 mmol, 1.00 equiv.) in DCM (10 mL) with N_2 bubbling was added DIEA (4.00 equiv.) dropwise and mixed for 2 h. Then MeOH (2.00 mL) was added to the resin with N_2 bubbling for 30 min. The resin was washed with DMF (100 mL * 5). Then 20% piperidine in DMF (40 mL) was added and the mixture was bubbled with N_2 for 30 min at 20 °C. The mixture was filtered to obtain the resin. The resin was washed with DMF (100 mL * 5) before proceeding to next step.

2) Coupling: A solution of DOTA-tri(t-butyl ester) (3.00 equiv.), HATU (2.85 equiv.) and DIEA (6.00 equiv.) in DMF (10 mL) was added to the resin with N_2 bubbling for 1 h at 20 °C. The coupling reaction was monitored by ninhydrin test, if it showed colorless, the coupling was completed. The resin was then washed with DMF (100 mL * 5).

3) After the last position completed, the resin was washed with DMF (100 mL * 5), MeOH (100 mL * 3) and then dried under vacuum.

Peptide Cleavage:

1) Cleavage cocktail (20% HFIP/DCM, 30 mL) was added to the flask containing the side chain protected peptide at room temperature and the mixture was stirred for 15 min, repeated for three times.

2) After filtration, the filtrate was concentrated under vacuum, then the residue was dissolved in MeCN/H_2O (v/v, 1/1, 100 mL), followed by lyophilization directly to give compound **3** (1.77 g, crude) as a colorless oil. It was used in the next step directly without purification.

(3) **Synthesis of compound 4**.

To a solution of compound **3** (900 mg, 0.992 mmol, 1.00 equiv.) and TFP (494 mg, 2.98 mmol, 3.00 equiv.) in ACN (2.00 mL) was added EDCI (571 mg, 2.98 mmol, 3.00 equiv.) at 20 °C. The reaction mixture was stirred at 20 °C for 1 h. LCMS indicated compound **3** was consumed and the desired

compound was detected. The reaction mixture was purified by prep-HPLC (A: 0.1% TFA in H_2O, B: ACN) directly to afford compound **4** (340 mg, 32.5% yield, 99.0% purity) as a colorless oil, confirmed by LCMS (MS cal.: 1055.5, MS observed: $[M+H]^+$ = 1056.7).

(4) **Synthesis of compound 5**.

Compound **4** was stirred in a solution of TFA/Tis/H_2O (v/v/v, 92.5/5.0/2.5, 10 mL) at 20 °C for 2 h. LCMS indicated compound **4** was consumed and the desired MS (Rt = 0.325 min, $[M+H]^+$ = 888.4) was detected. The mixture was concentrated under reduced pressure. The residue was purified by prep-HPLC (A: 0.1% TFA in H_2O, B: ACN) directly to afford compound **5** (170 mg, 59.4% yield, 96.7% purity) as a colorless oil, confirmed by LCMS (MS cal.: 887.3, MS observed: $[M+H]^+$ = 888.3).

(5) **Synthesis of compound 7**.

Peptide Synthesis: The peptide was synthesized using standard Fmoc chemistry.

1) Resin preparation: DMF (100 mL) was added to the vessel containing Sieber Resin (0.200 mmol, 0.540 g, 0.370 mmol/g) with N_2 bubbling for 30 mins. The resin was washed with DMF (40 mL * 5), followed by adding 20% piperidine in DMF (40 mL) and bubbled with N_2 for 30 mins at 20 °C for Fmoc deprotection. The mixture was filtered and the resin was washed with DMF (40 mL * 5) before proceeding to next step.
2) Coupling: A solution of Fmoc-D-Lys(Boc)-OH (281 mg, 3.00 equiv.), HATU (216 mg, 2.85 equiv.) in DMF (10 mL) was added to the resin with N_2 bubbling. Then DIEA (6.00 equiv.) was added to the mixture dropwise and bubbled with N_2 for 30 mins at 20 °C. The coupling reaction was monitored by ninhydrin test, if it showed colorless, the coupling was completed. The resin was then washed with DMF (40 mL * 5).
3) Deprotection: 20% piperidine in DMF (40 mL) was added to the resin and the mixture was bubbled with N_2 for 30 mins at 20 °C. The deprotection reaction was monitored by ninhydrin test, if it showed blue or brownish red, the reaction was completed. The resin was then washed with DMF (40 mL * 5).
4) Step 2~3 was repeated for the following amino acids elongation: Number # 2-10, in Table 1.
5) After the last position completed, the resin was washed with DMF (40 mL * 5), MeOH (40 mL * 3) and then dried under vacuum.

Table S1: The list of amino acids and the corresponding reagents used on SPPS.

#	**Materials**	**Coupling reagents**
1	Fmoc-D-Lys(Boc)-OH (3.00 equiv.)	HATU (2.85 equiv.) and DIEA (6.00 equiv.)
2	Fmoc-Glu(O*t*Bu)-OH (3.00 equiv.)	HATU (2.85 equiv.) and DIEA (6.00 equiv.)
3	Fmoc-Phe-OH (3.00 equiv.)	HATU (2.85 equiv.) and DIEA (6.00 equiv.)
4	Fmoc-Pen(Trt)-OH (3.00 equiv.)	HATU (2.85 equiv.) and DIEA (6.00 equiv.)
5	Fmoc-Leu-OH (3.00 equiv.)	HATU (2.85 equiv.) and DIEA (6.00 equiv.)
6	Fmoc-Thr(*t*Bu)-OH (3.00 equiv.)	HATU (2.85 equiv.) and DIEA (6.00 equiv.)
7	Fmoc-Asp(O*t*Bu)-OH (3.00 equiv.)	HATU (2.85 equiv.) and DIEA (6.00 equiv.)
8	Fmoc-Ser(*t*Bu)-OH (3.00 equiv.)	HATU (2.85 equiv.) and DIEA (6.00 equiv.)
9	Fmoc-N-Me-Arg(Pbf)-OH (3.00 equiv.)	HATU (2.85 equiv.) and DIEA (6.00 equiv.)
10	2-(Chloromethyl)benzoic acid (2.00 equiv.)	PyAop (2.00 equiv.) and DIEA (4.00 equiv.)

Peptide Cleavage, Cyclization and Purification:

1) Cleavage cocktail (TFA/3-MPA/Tis/H_2O (v/v/v/v, 92.2/2.5/2.5/2.5, 20 mL) was added to the flask containing the side chain protected peptide at room temperature and stirred for 1 h.
2) After filtration, the filtrate was precipitated with cold isopropyl ether and centrifuged (3 mins at 3000 rpm).
3) The crude peptide was washed with isopropyl ether for two additional times.
4) The crude peptide was dried under vacuum for 2 h to obtain compound **6** (crude). It was used in the next step directly without purification.
5) To a solution of compound **6** in MeCN/H_2O (v/v, 1/1, 200 mL) was added $NaHCO_3$ until pH reached ~8. Then the mixture was stirred at 20 °C for 2 h. LCMS showed compound **6** was consumed and desired MS (Rt = 0.285 min, $[M+H]^+$ = 1255.5) was detected. To the reaction mixture was added 1 M HCl until pH reached ~6. The mixture solution was dried by lyophilization to give crude compound **7**.
6) The crude peptide was purified by prep-HPLC (A: 0.1% TFA in H_2O, B: ACN) to afford compound **7** (166 mg, 66.7% purity, 66.4% yield) as a white solid, confirmed by LCMS (MS cal.: 1254.6, MS observed: $[M+H]^+$ = 1256.8).

(6) **Synthesis of compound 8**.

To a solution of compound **7** (162 mg, 129 μmol, 2.20 equiv.) and compound **2** (26.0 mg, 58.9 μmol, 1.00 equiv.) in DMF (1.5 mL) was added DIEA (60.9 mg, 471 μmol, 78.0 μL, 8.00 equiv.) at 20 °C. The mixture was stirred at 20 °C for 1 h. LCMS indicated compound **7** was consumed and desired MS (Rt = 0.358 min, $[M+2H]^{2+}$ = 1361.8) was detected. The mixture was precipitated with cold isopropyl ether and centrifuged (3 mins at 3000rpm). The crude peptide was washed with isopropyl ether for two additional times. The crude peptide was dried under vacuum for 2 h to obtain compound **8** (200 mg, crude) as a white solid, confirmed by LCMS (MS cal.: 2722.10, MS observed: $[M+2H]^{2+}$ = 1361.8). It was used in the next step directly without purification.

(7) **Synthesis of compound 9**.

Compound **8** (200 mg) was stirred in a solution of 50% TFA/DCM (10 mL) at 20 °C for 0.5 h. LCMS indicated compound **8** was consumed and desired MS (Rt = 0.335 min, $[M+2H]^{2+}$ = 1311.6) was detected. The mixture was concentrated under reduced pressure. The residue was precipitated with cold isopropyl ether and centrifuged (3 mins at 3000rpm). The crude peptide was washed with isopropyl ether for two additional times to crude peptide. The crude peptide was purified by prep-HPLC (A: 0.1% TFA in H_2O, B: ACN) directly to afford compound **9** (117.3 mg, 60.8% yield, 92.1% purity) as a white solid, confirmed by LCMS (MS cal.: 2621.9, MS observed: $[M+2H]^{2+}$ = 1311.6).

(8) **Synthesis of compound A2**.

To a solution of compound **9** (110 mg, 41.9 μmol, 1.00 equiv.) and compound **5** (41.0 mg, 46.1 μmol, 1.10 equiv.) in DMF (1.5 mL) was added DIEA (65.1 mg, 503 μmol, 84.0 μL, 12.0 equiv.) at 20 °C. The mixture was stirred at 20 °C for 1 h. LCMS indicated compound **9** was consumed and desired MS (Rt = 0.336 min, $[M+3H]^{3+}$ = 1115.3) was detected. The mixture was purified by prep-HPLC (A: 0.1% TFA in H_2O, B: ACN) directly to afford **A2** (10.6 mg, 7.57% yield, 95.2% purity) as a white solid, confirmed by LCMS (MS cal.: 3343.78, MS observed: $[M+3H]^{3+}$ = 1115.5) shown in **Figure S2**.

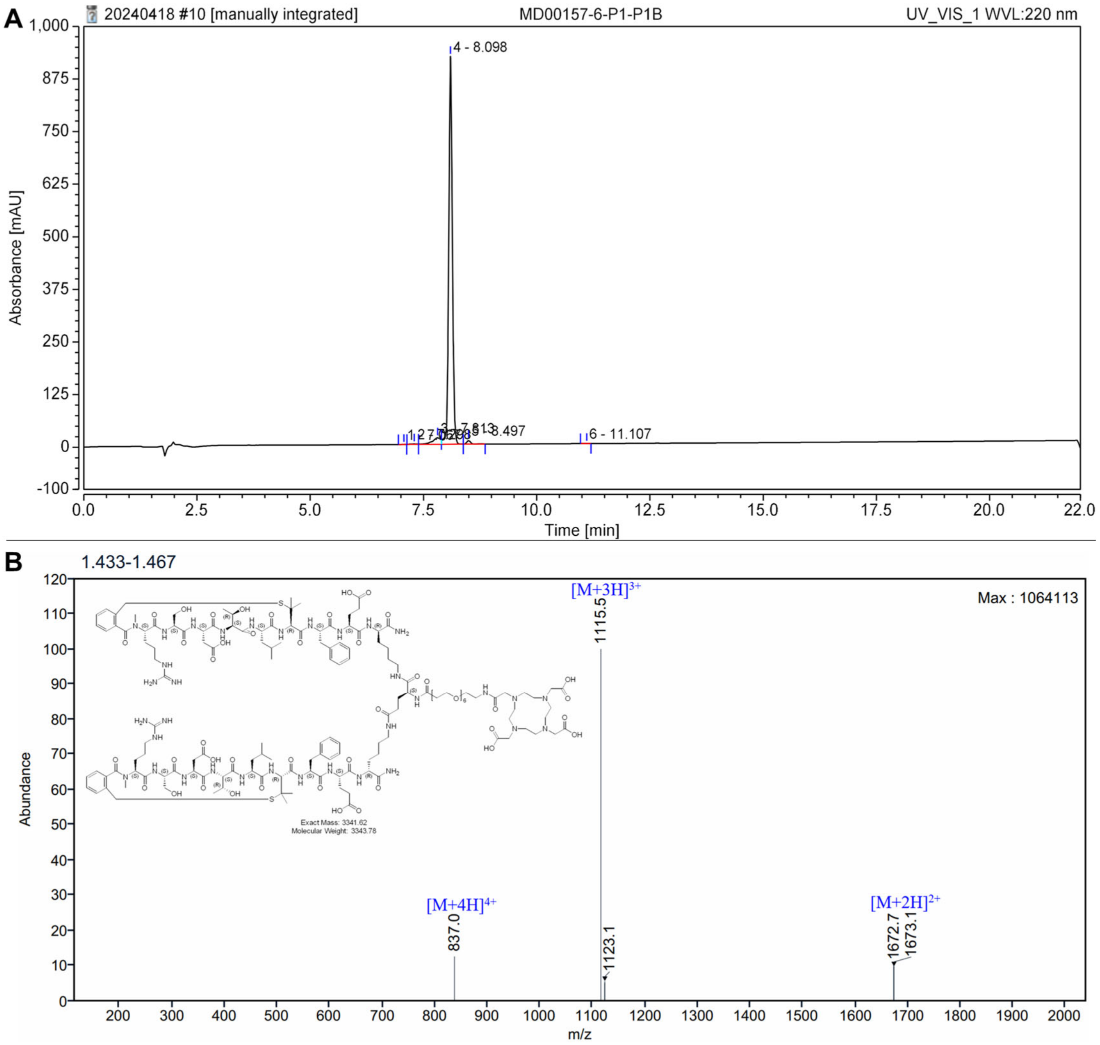

Figure S2. Representative HPLC chromatogram images and TOF MS results of A2.

2. Ex vivo imaging of colons of DAA-treated mice and healthy mice

For ex vivo imaging, the mice were sacrificed via cervical dislocation at 30 min post-injection and the colons were removed for PET/CT imaging directly without intestinal contents removal. A micro-PET (Siemens Inveon PET) was used for ex vivo imaging.

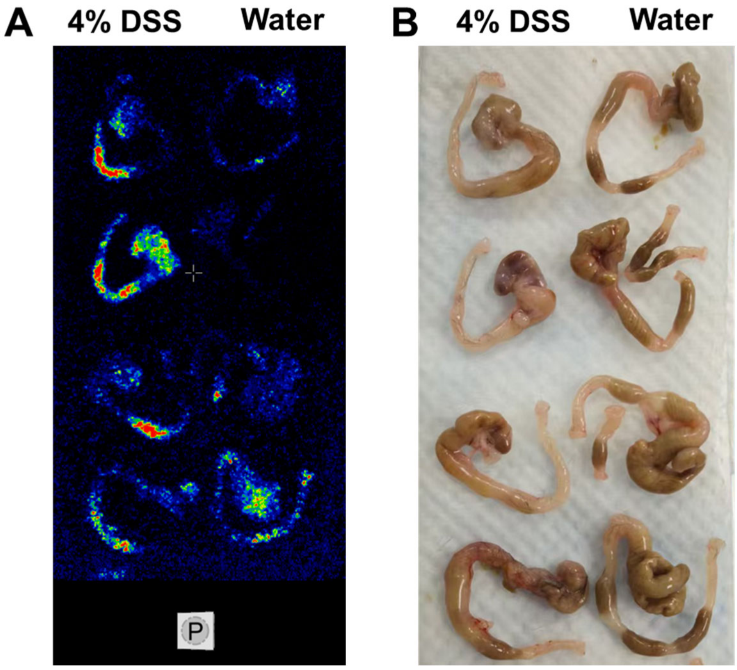

Figure S3. Ex vivo PET imaging of colons of DSS-induced mice and healthy mice at 30 min post-injection. （A）PET images of colons removed from DSS-induced mice (left panel) and healthy mice (right panel). （B）Bright field images of colons removed from DSS-induced mice (left panel) and healthy mice (right panel).

3. Acute toxicity study of ^{68}Ga-A2 in normal mice.

The acute toxicity experiment was conducted to evaluate the safety of ^{68}Ga-A2 by assessing whether intravenous administration induces drug-related acute adverse effects in mice, a prerequisite for clinical ethics application and translational research. 8 ICR mice with a bodyweight of 18-22 g were divided into two groups randomly. The mice were raised under normal feeding conditions before the experiment and throughout the observation period of the experiment. ^{68}Ga-A2 was prepared as required. For the ^{68}Ga-A2 group, each mouse was intravenously injected with 18.5 MBq/100 μL of ^{68}Ga-A2 at a uniform speed within 5 seconds. In the control group, 4 mice were intravenously injected with 100 μL of normal saline at a uniform speed within 5 seconds. No mice in the experimental group and the control group died within 24 hours. After 72 hours, the activities of the mice in both groups were normal, and there was no difference in body weight between the experimental group and the control group. Then all the mice were sacrificed and dissected. There was no obvious difference in the color and morphology of the organs of the mice in the experimental group compared with those in the control group.

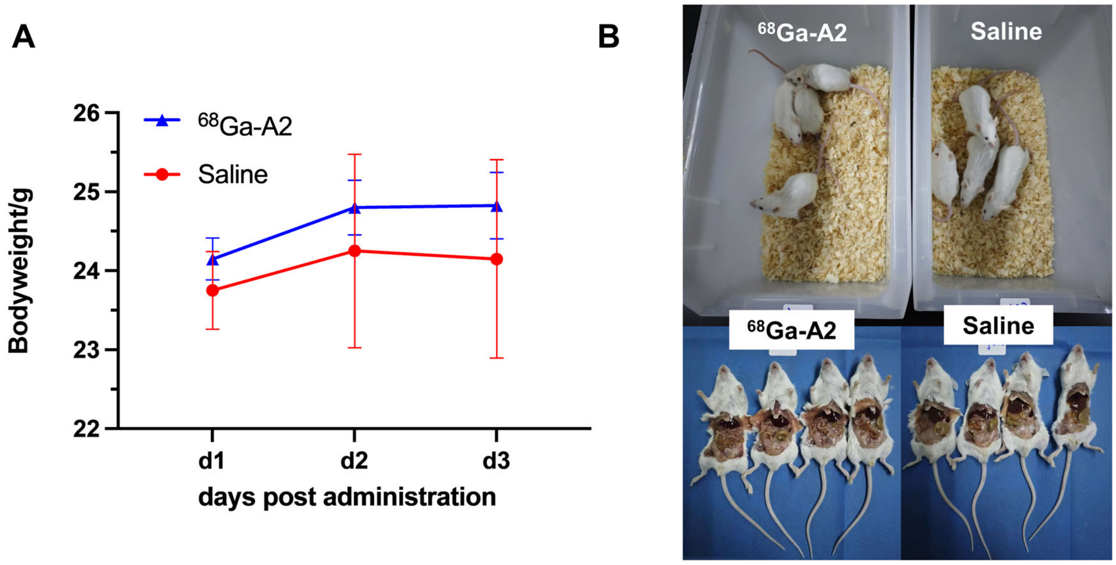

Figure S4. Acute toxicity study of ^{68}Ga-A2 in normal mice. (A) Bodyweight changes after injection of ^{68}Ga-A2 or saline. (B) The activity of mice and conditions of the organs of the mice.

4. Determination of *in vitro* and *in vivo* stability of ^{68}Ga-A2

For the in *vivo* stability studies, normal C57BL/6JNifdc mice (female, 6 weeks of age) were injected with ^{68}Ga-A2 (18 MBq) through the tail vein. Murine urine samples at 30 and 120 min post-injection were collected and mixed with acetonitrile, and then passed through a 0.22 μm Millipore filter. Then the samples were analyzed with radio-HPLC. For *in vitro* stability in mouse serum, ^{68}Ga-A2 (18 MBq) was incubated with fresh mouse serum for 30 or 120 min. Then the samples were mixed with acetonitrile, and then passed through a 0.22 μm Millipore filter. Then the samples were analyzed with radio-HPLC. Phase A was water with 0.1% TFA and phase B was ACN with 0.1% TFA. The flow rate was 1 mL/min. The gradient mobile phase started from 20% phase B and progressed to 80% phase B at 20 min. It should be noted that a new analytical column (InfinityLab Poroshell 120 EC-C18, 100 × 4.6 mml, 4 μm) was used for this assay because the original analytical column became unusable due to excessively high column pressure caused by long-term use. Although the retention time of 68Ga-A2 changes, the replacement of the column does not affect the conclusions of this experiment, as the peak profile exhibited by ^{68}Ga-A2 is consistent with those obtained previously. The stability results were shown in Figure S5.

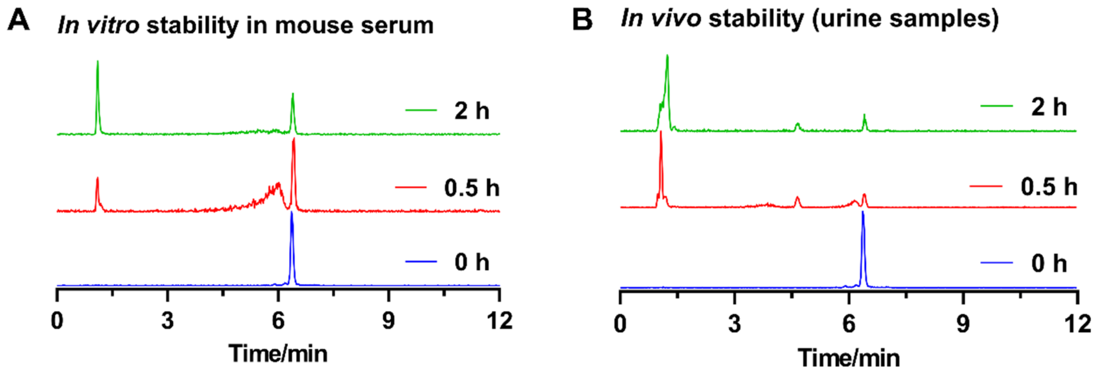

Figure S5. (A) *In vitro* stability of ^{68}Ga-A2 after incubation with mouse serum for 30 or 120 min. (B) In vivo stability of ^{68}Ga-A2 using mouse urine samples for analysis. The radiolabeling yield and radiochemical purity of ^{68}Ga-A2 was determined by radio-HPLC.
